# Intravenous acetaminophen for postoperative pain in the neonatal intensive care unit: A protocol for a pilot randomized controlled trial (IVA POP)

**DOI:** 10.1371/journal.pone.0294519

**Published:** 2023-11-20

**Authors:** Victoria Anne Archer, Samira Samiee-Zafarghandy, Forough Farrokyhar, Daniel Briatico, Luis H. Braga, J. Mark Walton

**Affiliations:** 1 Division of General Surgery, Department of Surgery, McMaster University, Hamilton, ON, Canada; 2 Division of Neonatology, Department of Pediatrics, McMaster University, Hamilton, ON, Canada; 3 Department of Surgery, McMaster University, Hamilton, ON, Canada; 4 Division of Urology, McMaster University, Hamilton, ON, Canada; 5 Division of Pediatric General Surgery, Department of Surgery, McMaster University, Hamilton, ON, Canada; Maulana Azad Medical College, INDIA

## Abstract

**Background:**

In neonates, uncontrolled pain and opioid exposure are both correlated with short- and long-term adverse events. Therefore, managing pain using opioid-sparing approaches is critical in neonatal populations. Multimodal pain control offers the opportunity to manage pain while reducing short- and long-term opioid-related adverse events. Intravenous (IV) acetaminophen may represent an appropriate adjunct to opioid-based postoperative pain control regimes. However, no trials assess this drug in patients less than 36 weeks post-conceptual age or weighing less than 1500 g.

**Objective:**

The proposed study aims to determine the feasibility of conducting a randomized control trial to compare IV acetaminophen and fentanyl to a saline placebo and fentanyl for patients admitted to the neonatal intensive care unit (NICU) undergoing major abdominal or thoracic surgery.

**Methods and design:**

This protocol is for a single-centre, external pilot randomized controlled trial (RCT). Infants in the NICU who have undergone major thoracic or abdominal surgery will be enrolled. Sixty participants will undergo 1:1 randomization to receive intravenous acetaminophen and fentanyl or saline placebo and fentanyl. After surgery, IV acetaminophen or placebo will be given routinely for eight days (192 hours). Appropriate dosing will be determined based on the participant’s gestational age. Patients will be followed for eight days after surgery and will undergo a chart review at 90 days. Primarily feasibility outcomes include recruitment rate, follow-up rate, compliance, and blinding index. Secondary clinical outcomes will be collected as well.

**Conclusion:**

This external pilot RCT will assess the feasibility of performing a multicenter RCT comparing IV acetaminophen and fentanyl to a saline placebo and fentanyl in NICU patients following major abdominal and thoracic surgery. The results will inform the design of a multicenter RCT, which will have the appropriate power to determine the efficacy of this treatment.

**Trial registration:**

ClinicalTrials.gov NCT05678244, Registered December 6, 2022.

## Introduction

Pain management in neonates is of significant clinical and ethical importance. Pain in the neonate began to garner academic interest in the 1980s when Anand et al. demonstrated that unmanaged pain is associated with hemodynamic instability, muscle and protein catabolism, intraventricular hemorrhage, and leukomalacia [1–4]. Furthermore, researchers determined that due to their rapid state of neurodevelopment, uncontrolled pain may lead to long-term neurodevelopmental changes and chronic pain [5–12].

As evidence highlighted the need to control pain in neonates adequately, the use of opioids in NICUs increased [13]. For the management of both operative and non-operative pain, opioids, especially fentanyl, remain the most common medication used [14, 15]. There is limited data about the frequency of use of opioids after surgery in the NICU. Authors have reported that between 88–93% of NICUs predominantly use opioids for the management of postoperative pain [16, 17]. In a retrospective chart review in 1997, Johnston et al. found that all 13 postoperative patients in their cohort received opioids for postoperative pain, most commonly via a continuous infusion [18]. Opioids can provide analgesia but are associated with adverse events. Acutely, they are associated with respiratory depression, urinary retention, hypotension and tolerance [19–24]. Similar to neonates’ response to uncontrolled pain, their state of neurodevelopment may make them susceptible to long-term adverse events, although more research is needed. The small-scale studies completed previously have identified that opioid exposure may be linked the reduced intelligence, memory, and social problems; however, it is unclear if these changes are reversible and persist into adolescence and adulthood [25–27].

The mounting evidence demonstrating the need to adequately manage pain while also reducing opioid use in neonates has led to an interest in the development of multimodal pain regimes [28]. These regimes are commonplace in managing postoperative pain in adults and older children [29–32]. These regimes, however, cannot simply be applied to the neonatal population. The unique physiology of neonates, particularly pre-term neonates, necessitates specific attention, including evaluating the efficacy of individual medications [33, 34].

### Rationale

One of the most promising non-opioid analgesics is IV acetaminophen. This medication is beneficial postoperatively as many patients cannot take medications orally or rectally, and it has more predictable responses in neonates than other routes [35]. We recently conducted a systematic review on IV acetaminophen for postoperative pain in pediatric patients recovering from abdominal and thoracic surgery. We found that when it is added to opioid-based pain regimes, there is a reduction in opioid use and minor adverse events without sacrificing pain control. However, we identified no trials that evaluated IV acetaminophen for postoperative pain in patients under 36 weeks gestational age or birth weight under 1500 grams. In pre-term neonates, slight differences in gestational age may significantly impact physiology. Therefore, the data from older children and neonates cannot be applied to this population. Furthermore, all available trials suffer from short follow-up periods, with the median follow-up time being 48 hours. Recovery after major surgery extends well beyond this time; prolonged follow-up times are needed to capture the entire postoperative period [36]. While there may be clinical benefits, IV acetaminophen is expensive. It is reported to be 20 times more expensive than rectal formulations and 84 times more expensive than oral liquid formulations [37].

IV acetaminophen has not been studied in pre-term infants; therefore, its clinical efficacy remains unknown. The research question of this study is: “Does the addition of IV acetaminophen to fentanyl-based postoperative pain regimes (compared to placebo) reduce opioid use and adverse events without sacrificing pain scores?” In order to determine whether its clinical effect is worth the significant cost, its efficacy must first be established. We therefore developed a study protocol to answer this question. This trial will be the first to examine the efficacy of IV acetaminophen for postoperative pain in pre-term neonates. It will also be the first to use a prolonged follow-up period. Our study will build on existing data and may uncover novel insights into the care of pre-term neonates in the extended perioperative period.

## Methods

### Trial design and objectives

This study is a single-center, blinded, parallel-arm, placebo-controlled, superiority, external feasibility randomized controlled trial. The primary objective of this pilot study is to determine the feasibility and cost of conducting a RCT to assess the effect of IV acetaminophen on postoperative pain in preterm neonates in the neonatal intensive care unit.

### Setting and timeline

This study will occur in the NICU at McMaster Children’s Hospital (MCH) in Hamilton, Ontario, Canada. This NICU has 72 beds and admits more than 1,500 infants annually. Using Canadian Neonatal Network data from 2018–2020 and local internal case recording, an estimated 41 eligible cases will occur each year. Assuming a recruitment rate of 60%, our estimated recruitment period will be approximately 2 years [38]. Recruitment opened March 20, 2023. Two patients have been recruited.

### Participants

The participants of this study will include neonates admitted to the NICU at MCH who have undergone major open abdominal or thoracic surgery (as defined by the Canadian Neonatal Network, S1 Table) [38]. Neonates of all corrected gestational ages will be eligible up until 12 months corrected gestational age. Patients who have or develop hepatic or renal dysfunction, as described in Table 1, will be excluded [39–44]. Neonates who received acetaminophen administration within 24 hours of the end of surgery (which includes intraoperative administration) will be excluded to prevent contamination groups. Neonates who have nerve blocks or epidurals will also be excluded. The medical team will be asked for approval of the patient to enter the study; if they have any clinical concerns (such as a duct-dependent cardiac lesion), the patient will be excluded. Patients can be enrolled up to 12 hours after surgery, including emergent overnight cases if the research team cannot obtain consent. If a patient is discharged from the McMaster NICU within the study period (including transfer to another institution, discharge home, or death), the patient will be prematurely withdrawn from the study, but data up to that point will be included. If a participant undergoes multiple surgeries during the recruitment period, they will only be eligible for inclusion once (i.e., if a patient was already enrolled, they may not be enrolled again for a second surgery).

**Table 1:** Inclusion and exclusion criteria for the IVA POP trial.

Inclusion Criteria	Exclusion Criteria
Neonates, admitted to McMaster Children’s Hospital NICU	Hepatic dysfunction • AST, ALT or Bilirubin > 3x upper limit of normal • INR ≥ 3.0 or PT greater than 20s regardless of vitamin K administration
Has had major open, thoracic or abdominal surgery (see additional document 1).	Renal dysfunction • Increase in serum creatinine ≥ 2x baseline (baseline: lowest value in first 5 days of hospitalization) • Urine output < 0.5 mL/kg/h for ≥ 12h
Informed consent obtained from guardians	Allergy or intolerance to acetaminophen or fentanyl
	Acetaminophen administration within 24 hours of the end of surgery
	Nerve blocks or epidurals
	Refusal or withdrawal of consent
	Enrolment in another competing trial
	More than 12 hours after the end of surgery
	12 months post conceptual age or greater in age
	Birthweight greater or equal to 2,500g.
	Discharged from the McMaster NICU
	Concern from medical team

### Interventions

Participants will be randomized in a 1:1 fashion to the treatment or control group. To maintain a pragmatic trial design and ensure that analgesia is provided when clinically indicated, all subjects in the treatment and control arms will receive fentanyl as per the standard of care and discretion of the NICU team. As in the NICU of MCH, a continuous fentanyl infusion is the drug of choice for postoperative pain management for all patients receiving major thoracic or abdominal surgery, with intermittent bolus dosing used at the discretion of the primary care team to optimize pain management. The specific dosing instructions for fentanyl (i.e., infusion or bolus, rate, frequency, the timing of escalation or de-escalation) for all the subjects in our study’s control or intervention arm will be directed by the NICU team. Dosing guidelines for infusions and boluses can be seen below in the fentanyl formulation section. We will maintain an accurate record of each subject’s fentanyl dosing in mcg/kg/day.

Subjects randomized to the treatment arm (acetaminophen and fentanyl) will receive the indicated dose of IV acetaminophen (including the age-appropriate loading dose if indicated) seen below in Table 2. Subjects randomized to the control (fentanyl) group will receive a saline placebo at the same time interval and volume as whichever dose of acetaminophen they would have received. The acetaminophen treatment dosing guidelines were developed in consultation with the neonatal pharmacists and neonatologists at MCH. The guidelines used at MCH are based on validated dosing guideline [45–49]. Should patients change age categories throughout the trial, their dose will be adjusted accordingly. The McMaster research pharmacy has calculated a maximum daily volume of administration (using the maximum gestational age and a weight of 3 kg) of 12 mL per day, and a maximum loading dose of 6 mL, meaning the maximum daily volume requirement would be 18 mL. Regardless of treatment assignment, this additional fluid will be accounted for in the patient’s total fluid intake to ensure they maintain an appropriate total fluid intake.

**Table 2:** IV Acetaminophen dosing guidelines for the IVA POP trial.

Current Gestational Age	Intravenous dose	Maximum daily dose
32+6 weeks and under	10 mg/kg IV q12h	22.5 mg/kg/day IV
33–36 weeks	Loading dose: 20 mg/kg IV x 1, then	30 mg/kg/day IV
	10 mg/kg IV q8h	
37+ weeks and less than ten days old	Loading dose: 20 mg/kg IV x 1, then 10 mg/kg IV q6h	40 mg/kg/day IV
37+ weeks and at least ten days old		
Pediatric dosing	Loading dose: 20 mg/kg IV x 1, then 10 mg/kg IV q6h	60 mg/kg/day IV
(44+ weeks and 28+ days old)		

The intervention will begin once the patient is returned to the NICU from the operating room or at the time of completion of the procedure if performed at the bedside. The fentanyl infusion will be assessed to be increased or decreased on an ongoing basis by the primary care team in keeping with current standard practice. Rescue doses of fentanyl will be provided in both groups at the discretion of the primary care team. Each dose will be recorded. Any additional analgesics provided by the primary care team will also be recorded.

Blood work (AST, ALT, Bilirubin, INR, PT, and creatinine) will be tested preoperatively and at least twice during the 192-hour follow-up period. Using data from the Hamilton Regional Laboratory Medicine Program, the volume of blood required for these tests is 2.6 mL. It will be done twice during the follow-up period (postoperative days 3 and 7) for a total of 5.2 mL. Liver and renal function tests are commonly required postoperatively. These tests will be done in a coordinated fashion with other required blood work to reduce the frequency and volume of blood draws. A meta-analysis of neonatal venous sampling concluded there is minimal risk with single blood draws of less than 5% blood volume and less than 10% over eight weeks [50]. A preterm neonate has a blood volume of approximately 100 mL/kg, so the volume of blood draws within this study complies with these suggestions, even in neonates weighing as little as 500 grams [51, 52]. Patients will be withdrawn from the study if there is evidence of hepatic or renal failure (as described in Table 1).

### Acetaminophen formulation

Avir Pharma brand IV acetaminophen will be used in this study. It will arrive from the supplier in 1000 mg/100 mL (10 mg/mL) IV bags. The appropriate amount of medication will be drawn from the bag using sterile technique and into a separate syringe; it does not require further dilution. It will be administered over 15 minutes. The research pharmacy will generate an appropriate label.

### Placebo formulation

Normal saline infusion will be used as the placebo. Like IV acetaminophen, it is clear and colourless, maintaining blinding. The research pharmacist will prepare a syringe with a volume corresponding to the IV acetaminophen dosing guidelines with an identical label to maintain blinding. It will also be administered over 15 minutes.

### Fentanyl formulation

Sandoz and Sterimax brand fentanyl will be used to prepare 20 mcg/mL, 5 mcg/mL and 1 mcg/mL (diluted in D5W, normal saline, or D10W) syringes to be used in infusion pumps as is standard practice in the MCH pharmacy and NICU. The pharmacist will select the concentration based on the ordered dose of the medication.

To prepare the drug, the pharmacy will send the raw syringes or vials of Sandoz or Sterimax brand fentanyl (50 mcg/mL). Using standard technique, the solution will be diluted and prepared to the final target concentration based on the patient’s required dose, as determined by the neonatologist. The solution will then be mixed, capped, and appropriately labelled. Infusion dosing (including escalation, de-escalation, and discontinuation) will be at the discretion of the NICU physician, with a usual dose ranging between 0–3 mcg/kg/h; however, some patients may require up to 5 mcg/kg/h [53]. The NICU physician will be eligible to provide bolus doses as needed. The dosing range for bolus fentanyl, as described by the International Evidence-Based Group for Neonatal Pain, is a slow IV push of 0.5 to 3 mcg/kg/dose every 2 for 4 hours, titrated to effectiveness [54]. The NICU physician will ultimately determine specific dosing on a case-by-case basis.

### Trial flow and follow-up

**Fig 1:**
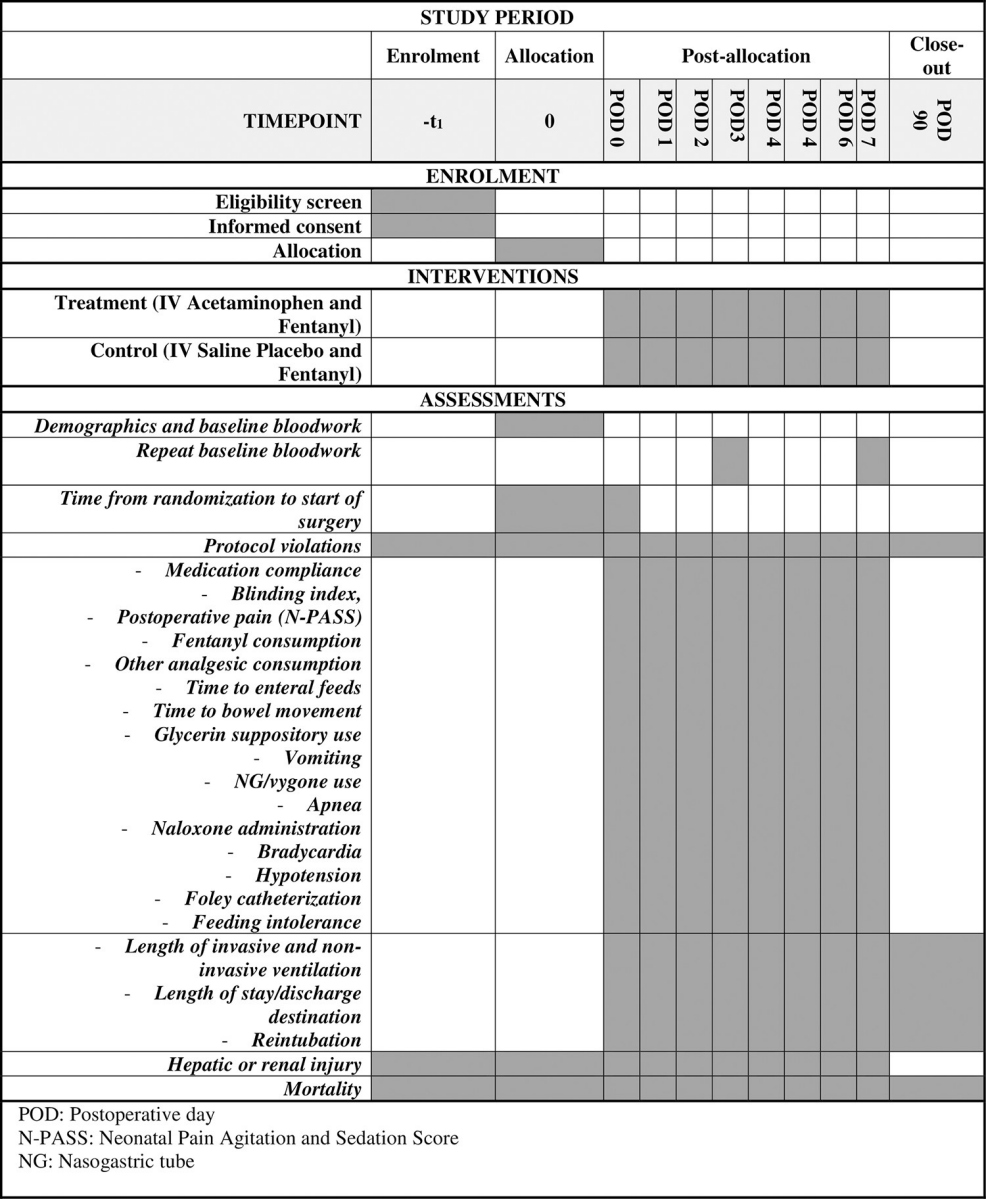
SPIRIT diagram representing the proposed schedule of enrolment, interventions, and assessments for the IVA POP trial.

Patients will receive the intervention from postoperative day zero to postoperative day seven for a total of eight days or 192 hours. Their postoperative day will be determined based on the time from the surgery’s end, as shown in Table 3. Data on opioid use, pain scores, and other clinical outcomes will be collected daily during these seven postoperative days. At the end of the week, cumulative opioid consumption and clinical outcomes will be calculated. At 90 days, a chart review will be done to assess the total length of assisted ventilation, length of stay, and mortality. Fig 1 below is a Standard Protocol Items: Recommendations for Interventional Trials (SPIRIT) diagram representing the proposed schedule of enrolment, interventions, and assessments for this trial. The SPIRIT checklist can be found in S1 Appendix. Feasibility outcomes related to trial recruitment rates are not included in this diagram but are described below.

**Table 3:** Time parameters for postoperative day designation for the IVA POP trial.

Post-Operative Day	Time From Surgery
Day 0	0–23 hours
Day 1	24–47 hours
Day 2	48–71 hours
Day 3	72–95 hours
Day 4	96–119 hours
Day 5	120–143 hours
Day 6	144–167 hours
Day 7	168–192 hours

### Sequence generation, randomization, and allocation concealment

Sequence generation and randomization will be done using Research Electronic Data Capture (REDCap), a secure, web-based software platform designed for study design and data capturing [55]. The allocation tables will be generated within REDCap by an unblinded, uninvolved research assistant. Patients will be randomized in a 1:1 fashion between the treatment and control groups. The sequence will be generated in randomly permuted blocks of four or six to ensure equal group sizes. Due to the small sample size, stratification will not be done; however, the balance of demographic features between groups will be evaluated as a feasibility outcome. The allocation tables will be provided in advance to the unblinded research pharmacists. Each participant will be assigned a study number. Using a linking key, the research pharmacy will be able to determine which arm the patient is randomized to. Medication will be prepared and dispensed by a trained research pharmacist. Only the research pharmacists, the unblinded research assistant and the data safety monitoring board members will have access to the randomization key. None of these parties are involved with data collection or analysis.

### Blinding and unblinding

Guardians, investigators, statisticians, nurses, allied health professionals and physicians will be blinded to treatment allocation. The research and clinical pharmacists will not be blinded as they will be involved in study drug preparation. They will not be blinded but will play no role in outcome assessment or analysis. Saline will be used for the placebo. Saline and IV acetaminophen are indistinguishable when prepared in solution. To ensure the protocol was followed, the key will be checked against the treatments provided at the end of the study. The patient’s chart will not mention which arm of the study the patient was enrolled in, therefore ensuring blinding at the 90-day chart review. The statistician will be provided with blinded data. The groups will not be revealed until after analysis.

If a safety concern is raised by the patient’s physician, member of the research team, the DSMB, the ethics committee, or Health Canada, the unblinded research assistant will be contacted with the patient’s name and participant number, and they will then provide their allocation. If unblinding occurs, the patient will be discontinued from the study.

### Data management and confidentiality

Study data will be collected and managed using REDCap [55]. Audit trails will be generated with this software. Any paper forms such as consents will be stored in a lock box in the secure Departments of Neonatology and Surgery; only study personnel will have the key to unlock this. Participants will be given a unique study identification number when randomized. The key linking study identification numbers to health record numbers will be kept as an encrypted Microsoft Excel file using AES-256 encryption software. Files will be stored on McMaster’s encrypted OneDrive platform. Identifying data collected in the study key include the patient’s medical record number, sex, date of birth, guardian’s name, and guardian’s contact information (phone, address, and e-mail). Data checks will be completed throughout the trial to ensure the accuracy of all data entered into REDCap. These checks will be completed after subjects 4, 12, and 20, with additional checks done randomly throughout the study. Following the trial, paper data will be maintained at McCulloch Office and Storage Systems in Hamilton, Ontario.

### Outcomes

Data to be collected are displayed below in Tables 4–7. When needed, additional elaboration is provided for key outcomes. Table 4 displays demographic variables to be collected.

**Table 4:** Demographic variables for IVA POP trial.

Sex
Race
Gestational age
Birth weight
Age at surgery
Preoperative diagnosis
Procedure
Length of OR
Preoperative opioid use (administered opioids within 7 days prior to surgery)
Intubated preoperatively
Maternal opioid use (use of opioids on more than two separate days during pregnancy)

**Table 5:** Primary outcomes for the IVA POP trial.

Outcome	Definition	Success
1. Monthly recruitment rate	Mean number of patients randomized per month	Average of 2 patients per month
2. Retention rate	Percentage of enrolled patients who fully complete the trial follow up	90% of patients enrolled followed completely
3. Medication compliance	Percentage of patients who received at least 80% of doses of study drugs at the correct dose and interval	80%
4. Blinding index [56].	Responses of nurse’s, medical staff, and research assistant’s guess of group assignment (control vs treatment) compared to actual group assignment	Less than 0.20

**Table 6:** Secondary feasibility outcomes for the IVA POP trial.

Outcome	Definition	Rationale
Time from randomization to start of surgery	Number of hours, positive hours indicate randomized prior to surgery, negative hours indicate randomized after surgery	This will help determine the average number of hours until surgery, to help determine how much lead time pharmacy must prepare and administrate medications.
Recruitment rate	Number of patients randomized/number of eligible patients	Will help estimate recruitment time in the largescale trial
Number of patients with one or more protocol violation	-	Assess feasibility of adhering to the current protocol
Cost	Canadian dollars per patient	Will help more accurately estimate the cost of a largescale RCT.
Amount of additional analgesics administered at each pain score in each arm.	Number of patients receiving rescue doses and dose administered (per weight) of additional analgesics for each pain score for pain scores above 6	Ensure that NICU physicians use of additional analgesics is balanced between the groups.

**Table 7:** Secondary clinical outcomes for the IVA POP trial.

Outcome	Definition
Postoperative Pain	N-PASS pain score every four or six hours, filled out by nurse
Fentanyl consumption	Cumulative over 24/hour periods, over entire study period, and duration of fentanyl infusion
Consumption of other analgesics	Cumulative over 24/hour periods and over entire study period
Invasive ventilation	Length of time requiring intubation
Non-invasive ventilation	Length of time requiring CPAP, BiPAP, or supplemental oxygen
Enteral feeds	Time to first enteral feeds and time to full enteral feeds (using NICU’s calculated goal feed)
Bowel movement	Time to first bowel movement
Glycerin suppository use	Number of patients requiring one or more glycerin suppositories
Length of stay	At 90-day chart review (with discharge destination)
Vomiting	Number of patients with ≥ 1 episode of vomiting documented
NG/Vygone	Number of patients, mean duration
Reintubation	Number of patients
Apnea	Number of patients with a documented oxygen saturation less than 94% or respiratory less than 20 breaths/min
Naloxone administration	Number of patients
Bradycardia	Number of patients with a documented heart rate less than 100
Hypotension	Number of patients with documented systolic blood pressure less than 60, or requiring vasoactive medication
Foley catheterization	Number of patients, mean duration
Feeding intolerance	Number of patients: feeds stopped or decreased due to vomit/increased gastric output, or if diagnosed by the treating team
Hepatic injury	Number of patients
Mortality	All-cause mortality at 90 days

### Feasibility outcomes

The primary outcomes of recruitment rate, retention rate, medication compliance, and blinding index have all been pre-selected to determine the success of the pilot RCT. The primary outcomes and the predetermined definition of success are displayed below in Table 5. For the trial to be deemed completely feasible each of these primary outcomes must meet the pre-specified success criteria. We will also include other outcomes to assess feasibility. Below in Table 6, we have described the secondary feasibility outcomes and their rationale and measurement.

### Secondary clinical outcomes

As this study is a pilot, it will not be adequately powered to assess these clinical outcomes fully; however, collecting these data will indicate the feasibility of data collection and may provide important preliminary data. The rationale and measurement for key secondary clinical outcomes are described below and displayed in Table 7.

### Pain scores

The primary outcome of the multicenter trial will be postoperative pain scores. Postoperative pain will be assessed using the pain component of the Neonatal Pain Agitation and Sedation Scale (N-PASS). N-PASS has two scorable components, sedation and pain. For this study we will only utilize the pain component of this score. Pain is scored on a scale of 0 to 13. It includes subjective assessments of behaviour and objective assessments of vital signs [57, 58]. The N-PASS pain score is validated in term and preterm neonates and for assessment of prolonged and postoperative pain. It is recommended by the Neonatal Pain-Control Group [57, 59, 60]. It has been independently validated and is reliable with high clinical utility [61, 62]. The N-PASS pain score is the measurement scale currently used in the MCH NICU. The bedside nurses, who will be responsible for measuring pain, are comfortable using this instrument, increasing the validity of the measurements. Furthermore, documenting this pain score regularly is part of their daily workflow. As this is already required documentation, the risk of missing data is reduced. It will reduce the burden of documentation required by the nurses.

Initial pain score will be recorded when the patient returns from the operating room. Pain scales will then be done just before the patient’s dose of acetaminophen or placebo (every 12, 8, or 6 hours), as recommended by the Neonatal Pain-Control Group [60]. The neonatologists will have access to the patient’s N-PASS score to aid in clinical decision-making.

### Statistical analysis

As this is a feasibility study, all comparative analyses on clinical outcomes are for exploratory purposes, and no inferences will be made from these analyses. Demographics will be reported as means or medians for continuous variables and frequencies and proportions for categorical variables. Feasibility outcomes (recruitment rate, completion rate, rate of patients discharged at 90 days, success of randomization, study related adverse events, and protocol violations) will be reported as proportions. Blinding success will be reported with the calculated blinding index, as described by James et al. [56]. Cost will be reported in Canadian dollars for the total cost and cost per participant randomized. Secondary clinical outcomes (pain scales, cumulative consumption of fentanyl and other drugs, number of rescue doses, length of time requiring fentanyl infusion, length of time requiring non-invasive (intubation, CPAP, BiPAP, supplemental oxygen), time to enteral fees, time to first bowel movement, and length of stay) will be reported as means or medians with standard deviation (SD) and interquartile ranges, respectively. As this study is not adequately powered to detect differences hypothesis testing will not be conducted. Statistics Package for the Social Sciences (SPSS) will be used for data analysis [63].

### Sample size

A sample size of 30 per arm (a total of 60 patients) will be used. This decision is based on methodologic guidelines, suggesting that this is ideal for assessing feasibility and calculating future sample sizes [64–66]. Daily communication with the surgical and medical teams will be used to achieve participant enrollment.

### Subgroup analysis

In the full RCT sub-group analysis is planned for the following demographic variables: gestational age (<32 weeks, ≥32 weeks), location of operation (thorax or abdomen), preoperative opioid use, and sex. Aside from sex, these have all been identified as predictors of postoperative pain [67–70]. Gestational age was selected rather than birth weight, as it is a better measure of the infant’s development and a more accurate predictor of physiologic response to pain [71].

### Safety and ethics

This study was approved by the Hamilton Integrated Research Ethics Board (14887), the Neonatal Research Committee at McMaster Children’s Hospital, and Health Canada (268629). Parents or legal guardians will provide written and informed consent prior to enrollment. The ethics approved protocol can be found in S2 Appendix.

A data safety monitoring board (DSMB) will be formed, including at least one neonatologist and one pediatric surgeon. They will meet when five patients have been enrolled and every six months after that. They will be provided with the study key to unblind data. They will meet within fifteen days after a possible study-related adverse event (non-life-threatening/non-fatal) and within 48 hours after a study-related life-threatening or fatal adverse event. The DSMB will create a summary report for the steering committee to categorize adverse events based on severity and relatedness to the study drug. The DSMB cannot recommend stopping early for benefit but may recommend stopping for harm if they observe significant safety concerns.

## Discussion

### Anticipated pitfalls

Due to the types of surgical conditions that affect neonates in the NICU, we anticipate several pitfalls that may affect recruitment and follow-up. Many neonates who require surgery are treated on an emergent basis, which can occur overnight or on weekends. There is also minimal lead time with high-acuity emergency surgery. These factors represent a challenge for enrollment as we may be unable to capture all after-hours cases due to financial and logistic restraints. The research team will be in close contact with the surgical and NICU teams to attempt to be aware of any potential cases as soon as possible to reduce this risk.

Again, the emergent nature of many neonatal surgical conditions (e.g., necrotizing enterocolitis) may affect recruitment. Renal and hepatic dysfunction are both exclusion and withdrawal criteria for this trial. In preterm infants, immaturity of the renal system and nephrotoxic events (i.e., congenital abnormalities, sepsis, hypovolemia, aminoglycoside antibiotics) put patients admitted to the NICU at risk for acute renal failure. The precise incidence is unknown, but studies have demonstrated rates between 6% and 24% [72–74]. In preterm infants, rates of cholestasis have been reported to be up to 24%. There will also be infants with non-cholestatic hepatic dysfunction. Factors such as lack of enteral feeds, prolonged parenteral nutrition, sepsis, and hepatoxic medications, which are more common in surgical populations, have been shown to increase this risk of hepatic dysfunction [75, 76]. The relatively high incidences of these conditions may impact recruitment. For recruited patients, whether their renal or hepatic dysfunction is related to IV acetaminophen administration, they will have to be prematurely withdrawn from the study.

The measurement of pain Is Ir potential pitfall. The measurement of pain In Itself is subjective [77]. This is attenuated in neonates who are unable to communicate verbally. This has the potential to inject subjectivity not only into the results of the pain scores themselves but also into the amount of opioids prescribed, as the administration of opioids depends on the clinician’s interpretation of the patient’s pain score. While this subjectivity is not wholly avoidable, we have employed strategies to mitigate it. Firstly, we have selected a pain score validated for this clinical situation. And already used on this unit, meaning that staff are already comfortable with its administration and interpretation. Secondly, proper randomization should help balance variation in pain assessment and medication administration between groups.

Mortality is another potential limitation. The overall mortality rate after surgery for preterm infants is 16.8%. This rate increases as gestational age decreases with a postoperative mortality rate of 31% in patients less than 24 weeks gestational age [78]. Patients may die at any point along the study pathway, impacting recruitment and post-randomization withdrawal rates.

Loss to follow-up is unlikely as study participants will be monitored closely in the NICU and will not be discharged or transferred to another institution within eight days of major surgery. To enhance recruitment, we will continue proactively screening all potential participants. To reduce the frequency of post-randomization withdrawals, we will collect all data up until the point of withdrawal.

### Anticipated strengths

There are several methodologic features of this study which are anticipated strengths. First, the pragmatic design, which allows neonatologist control over other analgesics means the results of this RCT will be readily applicable to clinical practice. Furthermore, we purposely designed the trial to not significantly add to the workflow of the clinical team, which will reduce missing or incomplete data. The extended follow-up period is another strength of this trial. The primary 192-hour (eight-day) and secondary (90-day) follow-up periods will provide novel data on the extended perioperative period and may offer insights into the ideal duration of treatment and the longer-term ramifications of this intervention. This will be the first trial on this topic to have a follow-up period beyond 72 hours. This will also be the first trial to include pre-term neonates, which will offer new insights into the effect of this drug on this population [36]. Using James et al.’s blinding index as a feasibility outcome is another strength [56]. This measure was selected because we were concerned the efficacy of IV acetaminophen for pain control may lead to inadvertent unblinding of the clinical team at clinical team, and that bedside nurses in particular may be able to ascertain treatment assignment. This information will be essential in designing the multicenter RCT.

### Conclusion

There are no data evaluating the efficacy of IV acetaminophen for postoperative pain in preterm infants following major abdominal and thoracic surgery. This external pilot study will allow us to assess the feasibility of a superiority, multi-centred, placebo-controlled, parallel-design, randomized controlled trial. When completed, the multicentre study will be powered to determine if the addition of IV acetaminophen to fentanyl-based postoperative pain regimes reduces opioid exposure and adverse events without sacrificing pain scores.

### Trial status

Enrollment began on March 20, 2023, and is anticipated to be completed in September 2024. Results will be published in a relevant academic journal and on ClinicalTrials.gov following this. Protocol amendments will be communicated to the Hamilton Integrated Research Ethics Board and Health Canada. The trial registry will also be updated as required.

## Supporting information

S1 Appendix SPIRIT checklist. (DOC)

S2 Appendix Ethics approved protocol. (DOCX)

S1 Table Major abdominal and thoracic operations as defined by the Canadian Neonatal Network. (DOCX)

S1 File (DOCX)
