# Supplementary material for: Intravenous acetaminophen for postoperative pain in the neonatal intensive care unit: A protocol for a pilot randomized controlled trial (IVA POP)
Source: PLoS One. 2023 Nov 20;18(11):e0294519. doi: 10.1371/journal.pone.0294519 (PMC10659208; doi:10.1371/journal.pone.0294519)
Supplement: S2 Appendix — (DOCX) [file pone.0294519.s002.docx]

**1.A: Title**

Study Title: IVA POP: Intravenous Acetaminophen for Postoperative Pain in the Neonatal Intensive Care Unit: a Pilot Randomized Controlled Trial

**1.A.1: Research Team**

Resident PI: Dr. Victoria Archer

Staff PI: Dr. Mark Walton

Steering Committee: Dr. Samiee-Zafarghandy, Dr. Forough Farrokhyar, Dr. Luis Braga

Research Assistants: Mr. Daniel Briatico, Ms. Heather Johnston

Statistician: Office of Surgical Research

Sponsor: Hamilton Health Sciences Corporation

Peron(s) authorized to sign protocol and protocol amendments: Dr. Victoria Archer and Dr. Mark Walton

Version 14.3, June 6, 2023

**1.B Abstract**

**Purpose:** Opioid use in neonates is associated with short and long-term adverse events. Multi-modal pain control offers the ability to control pain while reducing opioid exposure. This topic has been relatively unexamined in preterm and term neonates. Specifically, this trial aims to evaluate the effect of adding IV acetaminophen to standard opioid-based pain regimes in neonates in the neonatal intensive care unit (NICU) undergoing major abdominal and thoracic surgery.

**Objectives:** The primary aim of the proposed study is to determine the feasibility and cost of conducting a multicenter, randomized control trial to compare the efficacy of IV acetaminophen and fentanyl, to fentanyl and saline placebo, in terms of reduction of postoperative pain, opioid use, adverse events. The primary outcome is feasibility; secondarily, efficacy and safety will be assessed.

**Design:** This single-center, parallel-arm, placebo-controlled, fully blinded, randomized controlled external feasibility trial will enroll patients admitted in the neonatal intensive care unit (NICU) who have undergone major, thoracic, or abdominal surgery. Patients will be randomized 1:1, with parallel allocation to receive acetaminophen and fentanyl or fentanyl and saline placebo. All study staff, clinical staff and guardians will be blinded. As this is a pilot study, no sample size will be calculated, however; we aim to enroll 60 patients.  A sample size calculation will be completed for the full RCT if it is deemed feasible from the results of this study. Patients will be followed for the day of surgery and the following 7 postoperative days (192 hours) and have their charts reviewed at 90 days.

**Impact:** The results of this study will be used to determine the feasibility of conducting a multi-center RCT to assess the effect of IV acetaminophen on fentanyl infusions in postoperative neonatal patients. The effect of IV acetaminophen for postoperative pain in preterm neonates has yet to be studied, this trial would generate novel insights into its efficacy. The prolonged follow-up period would also provide novel insights into recovery throughout the entire perioperative period.

**2.A Introduction**

Due to their variable stages of maturity, and their inability to communicate the management of postoperative pain in neonates represents a significant challenge for clinicians. Despite this, compared to older children and adults there has been limited research on postoperative pain management in this population [1-5]. Opioids are commonly used after surgery in neonates, however, due to their state of growth and development, neonates are uniquely at risk of short- and long-term side effects of opioid use [6]. Acutely, neonates experience similar physiologic changes as their adult counterparts, such as decreased respiratory drive, delayed intestinal motility, and sedation, leading to increased lengths of stay [7, 8]. Long-term, neonatal patients are uniquely vulnerable as they grow and develop, putting them at high risk for long-term developmental adverse events when exposed to opioids at a young age [9, 10]. Managing pain in preterm and term neonates is essential, as unmanaged pain can lead to impaired ventilation, circulatory changes, intraventricular hemorrhage, and periventricular leukomalacia, but it must be balanced against the risk’s opioids pose [9, 11-13]. A multimodal strategy provides the opportunity to balance pain control and medication-related adverse events by reducing the consumption of opioids.

Intravenous (IV) acetaminophen appears as a promising addition to neonatal postoperative pain management. Acetaminophen is available in rectal, oral, and IV formulations. In neonates, particularly those who are premature, gastric emptying time increases, the mucosa is in varying stages of maturity, resulting in decreased intestinal absorption. Similarly, the immature enterohepatic circulation decreases absorption, as does premature expulsion with bowel movements. IV acetaminophen’s direct entry to the circulatory system results in a more predictable response in neonates when compared to oral and rectal formulations [14]. IV administration also avoids first-pass metabolism, resulting in up to 50% less accumulation in the liver and decreased production of hepatotoxic metabolites [15]. IV acetaminophen is well tolerated, with multiple studies demonstrating safety[16]. Aside from pain management, it is used frequently for the closure of patent ductus arteriosus in preterm neonates, and again has been shown to be safe [17].

In our pre-publication systematic review and meta-analysis, we have found a paucity of data related to IV acetaminophen in pediatric patients. The data is heterogeneous and of overall low quality. When aggregated, the five relevant trials demonstrated little to no difference in postoperative pain scores when opioids alone were compared to opioids with IV acetaminophen ( -0.20, 95% CI -0.76 to 0.35, p 0.47). There may be a decrease in opioid consumption with the addition of IV acetaminophen to opioid based pain regimes (-1.95 oral morphine equivalent doses/kg/48h, 95% CI -3.95 to 0.05, p 0.06). There was also a decrease in minor adverse events (RR 0.39, 95% CI 0.11 to 1.43, p 0.15). This may indicate that pain control can be maintained with less exposure to opioids [18-22]. No trials evaluated IV acetaminophen for postoperative pain in patients under 36 weeks gestational age or less than 1500g. In preterm neonates, slight differences in gestational age have significant ramifications in physiology. Therefore, the data from older children and neonates cannot be applied to this population. Furthermore, all the trials described suffer from short follow-up periods (median 48 hours), recovery after surgery extends well beyond this time. This trial would be the first to examine the postoperative use of IV acetaminophen in preterm neonates and would also be the first to use a prolonged follow-up period. We will build on existing data and uncover novel insights into the care of preterm neonates in the extended perioperative period.

**2.B Objectives**

The primary objective of this pilot study is to determine the feasibility and cost of conducting a randomized control trial (RCT) to assess the effect of IV acetaminophen on postoperative pain in preterm neonates in the neonatal intensive care unit. Secondarily we will collect efficacy and safety outcomes.

**3.A Trial Design and Background Methods**

Single-center, fully blinded, parallel-arm, placebo-controlled, external feasibility randomized controlled trial. The trial will be conducted in compliance with the protocol, Health Canada’s guidelines for good clinical practice (GCP), and Hamilton Health Science’s regulatory requirements.

**4.A Eligibility**

Table 1 below displays the inclusion and exclusion criteria. A detailed list of major surgeries as defined by the Canadian Neonatal Network (CNN) can be found in appendix 1, table 6 [23]. Pre-existing hepatic dysfunction was defined using a neonatal adaptation of the Pediatric Acute Liver Failure Study Group guidelines (INR > 3.0 or PT greater than 20 seconds regardless of vitamin K administration), and using other commonly used biochemical benchmarks (AST, ALT, or Bilirubin > 3 times the upper limit of normal) [24-28]. Renal dysfunction was defined using the Kidney Disease Improving Global Outcome’s groups criteria [29]. Aspartate aminotransferase (AST), alanine transaminase (ALT), bilirubin, international normalized ratio (INR) and prothrombin time (PT), creatinine and urine output will be measured throughout the study, and should patients develop either condition they will also be withdrawn from the trial. Acetaminophen administration within 24 hours of the end of surgery (which includes intraoperative administration) is listed as an exclusion criterion to prevent contamination of the groups. Tachyphylaxis is not a concern with acetaminophen administration, therefore, other preoperative exposure will not be considered for exclusion [30]. Patients will be allowed to be enrolled up to 12 hours after the end of surgery to allow for the inclusion of emergent overnight cases in the event the research team is not available to obtain consent overnight. There will be no lower limit on age or weight included to capture all premature and very low birth weight infants. We will exclude patients that are over a year old, or patients whose birth weight is more than 2500 grams which will ensure that our population is representative of typical NICU patients. If a patient is discharged from the McMaster NICU within the study period (including transfer to another institution, discharge home, or death), the patient will be prematurely withdrawn from the study, but data up to that point will be used. If a participant undergoes multiple surgeries during the recruitment period, they will only be eligible for inclusion to the trial for once (i.e. if a patient was already enrolled, they may not be re-enrolled at a later time for a second surgery). The medical team will be asked for approval of the patient to enter the study; if they have any clinical concerns (such as a duct-dependent cardiac lesion), the patient will be excluded. {Palmer, 2008 #11045;Davidson, 2021 #20295}

| **Table 1**. Inclusion and Exclusion Criteria | |
| --- | --- |
| **Inclusion Criteria** | **Exclusion Criteria** |
| Neonates, admitted to McMaster Children’s Hospital NICU | Hepatic dysfunction   - - AST, ALT or Bilirubin > 3x upper limit of normal   - INR ≥ 3.0 or PT greater than 20s regardless of vitamin K administration |
| Has had major open, thoracic or abdominal surgery (see appendix 1, table 6). | Renal dysfunction   - Increase in serum creatinine ≥ 2x baseline (baseline: lowest value in first 5 days of hospitalization) - Urine output < 0.5 mL/kg/h for ≥ 12h |
| Informed consent obtained from guardians | Allergy or intolerance to acetaminophen or fentanyl |
|  | Acetaminophen administration within 24 hours of the end of surgery |
|  | Nerve blocks or epidurals |
|  | Refusal or withdrawal of consent |
|  | Enrolment in another competing trial |
|  | No later than 12 hours after the end of surgery |
|  | 12 months post gestational age or greater in age |
|  | Birthweight greater or equal to 2,500g. |
|  | Discharged from the McMaster NICU |
|  | Concern from medical team |

**4.A.1 Withdrawal**

If any of the criteria outlined in table 2 are met, patients will be withdrawn from the trial.

| **Table 2.** Withdrawal Criteria |
| --- |
| Guardian withdrawal of consent |
| Clinical team recommends discontinuation of trial |
| Criteria for hepatic or renal dysfunction (Table 1) are met |
| Patient is unable to tolerate treatment due to allergy or local or systemic intolerance |
| Discharge from the McMaster NICU |

If withdrawn from the trial, all outcomes until the point of withdrawal will be used, unless otherwise specified by the subject’s guardian. If withdrawn after administration of study medication, subjects will undergo the next repeat baseline blood work assessment as a safety assessment, however, this data will not be used for research purposes. Should this bloodwork be abnormal, the subject’s medical team will continue appropriate management.

**4.B Setting and Timeline**

This study will occur in the Neonatal Intensive Care Unit (NICU) at McMaster Children’s Hospital (MCH) in Hamilton, Ontario, Canada. This NICU has 72 beds and admits more than 1,500 annually. Using Canadian Neonatal Network (CNN) data from 2018-2020, an average of 2.85% of neonates were reported as requiring a laparotomy or thoracotomy each year. Based on the number of neonates admitted to MCH NICU annually, this leads to an estimated recruitment period of approximately 1.5 years [23].

**5.A.1 Interventions**

Enrolled subjects will be randomized to treatment or control group. To maintain a pragmatic trial design, and to ensure that analgesia is being provided when clinically indicated, all subjects in the treatment *and* control arms will receive fentanyl as per the standard of care and discretion of the NICU team. As in the NICU of McMaster Children’s Hospital (MCH), a fentanyl infusion is the drug of choice for postoperative pain management for all patients receiving major thoracic or abdominal surgery, with intermittent bolus dosing used at the discretion of the primary care team to optimize pain management. The specific dosing instructions for fentanyl (infusion or bolus, rate, frequency, timing of escalation or de-escalation, etc) for all the subjects in the control or intervention arm of our study will be directed by the NICU team. Dosing guidelines for infusions and boluses can be seen below in section 5.A.5 Fentanyl Formulation. We will maintain an accurate record of each subjects’ fentanyl detailed dosing instruction. Subjects randomized to the treatment arm (acetaminophen) will receive the indicated dose of IV acetaminophen (including the age-appropriate loading dose) seen below in Table 3. Subjects randomized to the control (fentanyl) group, will receive a saline placebo at the same time interval and volume as whichever dose of acetaminophen they would have received. The acetaminophen treatment dosing guidelines were developed in consultation with the neonatal pharmacists and neonatologists at McMaster Children’s Hospital. The guidelines used at McMaster Children’s hospital are based on validated dosing guidelines including the Stockholm Protocol and the associated guidelines as recommended by Lexicomp and UptoDate [31-35]. Should a patient change age categories throughout the trial their dose will be adjusted accordingly. The McMaster research pharmacy has calculated a maximum daily volume of administration (using the maximum gestational age and a weight of 3kg) of 12mL per day, and a maximum loading dose of 6mL, meaning the maximum daily volume requirement would be 18mL. Regardless of if this patient is in the placebo or control arm this additional fluid will be accounted for in the patient’s total fluid intake to ensure they continue to receive appropriate volumes of fluid.

| **Table 3.** IV Acetaminophen dosing guidelines | | |
| --- | --- | --- |
| *Current Gestational Age* | *Intravenous dose* | *Maximum daily dose* |
| 32+6 weeks and under | 10 mg/kg IV q12h | 22.5 mg/kg/day IV |
| 33-36 weeks | Loading dose: 20 mg/kg IV x 1, then  10 mg/kg IV q8h | 30 mg/kg/day IV |
| 37+ weeks and less than ten days old | Loading dose: 20 mg/kg IV x 1, then  10 mg/kg IV q6h | 40 mg/kg/day IV |
| 37+ weeks and at least ten days old |  |  |
| Pediatric dosing  (44+ weeks and 28+ days old) | Loading dose: 20 mg/kg IV x 1, then  10 mg/kg IV q6h | 60 mg/kg/day IV |

The intervention will begin once the patient is returned to the NICU from the operating room (OR) or at the time of completion of the procedure if performed at the beside. The fentanyl infusion will be assessed to be increased or decreased on an ongoing basis by the bedside nurse and primary care team in keeping with current standard practice. Rescue doses of fentanyl will be provided in both groups at the discretion of the primary care team. Each dose will be recorded. Any additional analgesics provided by the primary care team will also be recorded. Acetaminophen will be discontinued if there is evidence of hepatic failure (as described in table 1). AST, ALT, Bilirubin, INR, PT and creatinine will be tested preoperatively and at least twice during the 196-hour follow-up period. The patient will be withdrawn from the study if they meet the criteria for hepatic or renal failure as described in table 1. Using data from the Hamilton Regional Laboratory Medicine Program, the volume of blood required for these tests is 2.6 mL. It will be done twice in the follow up period (POD 3 and 7), for a total of 5.2 mL. Liver and renal function indices are a commonly required postoperative laboratory blood work in the NICU. When possible, it will be done opportunistically with other required blood work to reduce the frequency and volume of blood draws. A meta-analysis on neonatal blood draws concluded there is minimal risk with single blood draws of less than 5% blood volume, and less than 10% over 8 weeks [36]. With the average blood volume of a preterm being approximately 100mL/kg, the volume of blood draws within this study is compliant with these suggestions, even with neonates weighing 500g [37, 38]. When enrolled in the study, patients will not be able to have oral or rectal acetaminophen, nerve blocks or epidurals. Figure 1 demonstrates the flow of patients through the trial from identification to completion of the trial.


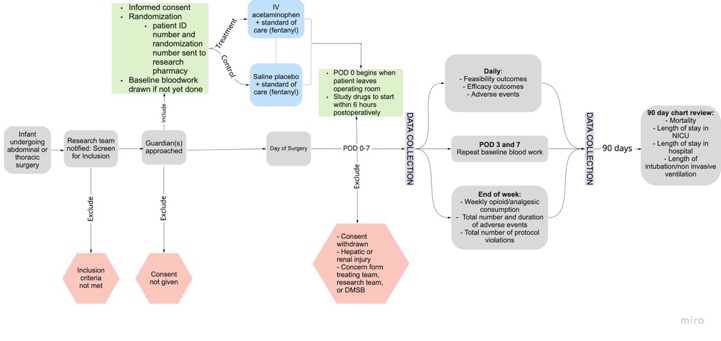


**Figure 1.** Flow of patients through the IVA POP NICU trial. POD: Postoperative Day

**5.A.2 Pre-screening and Baseline Evaluation**

Patients will be identified by the bedside nurse, the NICU medical team, or the surgical team when a patient has been consented for surgery. A research associate will then ensure all inclusion and exclusion criteria are met and approach the guardian(s) for consent. There will be no pre-operative medication or optimization. Orders will be written to ensure that patients do not receive acetaminophen within 24-hours of surgery, and to ensure that no regional anesthesia (i.e., nerve blocks or epidurals) are used intraoperatively. If the attending anesthesiologist feels these interventions are in the best interest of the patient the patient will be withdrawn from the study.

Baseline bloodwork will be done if not done within the last 5 days, and will include AST, ALT, ALP, Bilirubin, INR, PT, and Creatinine. If any of the exclusion criteria described above are met, the patient will be not included in the study. This baseline bloodwork will be repeated on postoperative day 3 and 7. Baseline characteristics including sex, race, gestational age, birthweight, maternal opioid use (use of opioids on more than two separate days during pregnancy), preoperative opioid use (administered opioids within 7 days prior to surgery), and preoperative diagnosis will also be collected at this time.

**5.A.3 Acetaminophen Formulation**

Avir Pharma brand IV acetaminophen will be used in this study. It will arrive from the supplier in 1000 mg/100mL (10 mg/mL) IV bags. The appropriate amount of the drug will be withdrawn from the bag using clean technique and into a separate clean syringe, it does not require further dilution. It will be administered over 15 minutes. An appropriate label will be generated by the research pharmacy. Further details of Avir Pharma brand IV acetaminophen can be seen in the investigator’s brochure.

**5.A.4 Placebo Formulation**

Normal saline will be used as the placebo. Like IV acetaminophen it is clear and colourless, maintaining blinding. The research pharmacist will prepare a syringe with a volume corresponding to the IV acetaminophen dosing guidelines with an identical label to maintain blinding. It will also be administered over 15 minutes.

**5.A.5 Fentanyl Formulation**

Sandoz and Sterimax brand fentanyl will be used to prepare 20 mcg/mL, 5 mcg/mL and 1 mcg/mL (diluted in D5W, normal saline, or D10W) syringes to be used in infusion pumps as is standard practice in the MCH pharmacy and NICU. The pharmacist will select the concentration based on the ordered dose of the medication. Please see Table 12 in Appendix 1 for more details.

To prepare the drug the pharmacy will send the raw syringes or vials of Sandoz or Sterimax brand fentanyl. Using standard clean technique, the solution will be diluted and prepared to the final target concentration based on the patient's required dose, as determined by the neonatologist This solution will then be mixed, capped, and appropriately labeled. Infusion dosing (including escalation, de-escalation, and discontinuation) will be at the discretion of the NICU physician, with a usual dose ranging between 0-3 mcg/kg/h, although some patients may require up to 5 mcg/kg/h [39]. The NICU physician will be able to provide bolus dosing as described in section 5.A.1 Intervention. Specific dosing will be determined on a case by case basis by the NICU physician, but are based on the International Evidence-Based Group for Neonatal Pain recommendations of a slow IV push of 0.5 to 3 mcg/kg/dose every 2 for 4 hours, titrated to effectiveness [40]. More detailed information on the formulation of fentanyl can be seen in Appendix 2, figure 2.

**5.A.6 Consent**

A member of the research team will obtain informed consent from the patient’s guardian. This will be done separately from the surgical consent so that guardian’s do not feel that their surgical management is contingent on their child’s participation in the trial. Languangeline will be used for translation purposes in the event the guardian does not speak English.

**6.A Primary Outcome Measures:** The primary objective of this trial is to evaluate the feasibility of conducting a full RCT. Recruitment rate, follow up rate, medication compliance, and blinding index (ratio of observer assessment of each participants group assignment (treatment vs. control) compared to actual assignment) have all been pre-selected to determine the success of this trial.

1. Recruitment rate: average rate of at least 2 patients per month

2. Follow up rate: 90% of patients followed for entire follow up period (postoperative day 0-7).

3. Compliance: 80% of patients receiving 80% of doses of study drug at correct dose and interval [41].

4. Blinding index (for nurses, physicians, and research assistants): Less than 0.20 (as predefined by James et al) [42].

Specifically, James et al blinding index was selected as the clinical experience of the bedside nurses and physicians in the NICU means that there is a risk that they will be able to determine which treatment a patient is receiving based on physiologic responses, despite efforts to blind them. The blinding success rate will be evaluated by asking the bedside nurse at the end of each shift, and physicians and data collection research assistants daily to indicate if they felt their patient was randomized to the treatment group, the control group, or if they were unsure. Physicians will similarly complete this index once daily during rounds. These results will be compared to the actual allocation using a blinding index [42].

**6.A.1 Secondary Feasibility Outcomes:**

The time from randomization to start of surgery will be recorded to determine the average number of hours until surgery, to help determine how much lead time pharmacy has to prepare and administrate medications. The average cost per patient will be calculated by dividing the final cost of the trial per number of patients enrolled. We will also compare additional analgesic use between the control and intervention arms for patients with similar pain scores. We have elected to maintain a pragmatic trial design and allow neonatologists to provide additional analgesia as they feel is clinically indicated. This decision may introduce bias, therefore this result will aide in determining if more structured guidelines are required for our future multi-center RCT. Should there be significant difference between pain scores and fentanyl administration between the arms this may indicate the need for more structure. For a comprehensive list and definitions of feasibility outcomes refer to appendix 1 table 8.

**6.B Secondary Outcome Measures:** The secondary objectives of this study are related to clinical efficacy and adverse events. The efficacy outcomes of interest are postoperative pain scores, cumulative fentanyl use (over 24 hours, over entire study period), duration of fentanyl infusion, number of fentanyl rescue doses, consumption of over analgesics and sedatives (over 24 hours, over entire study period, and number of rescue doses), invasive ventilation requirements (length of time), non-invasive ventilation requires (CPAP, BiPAP, supplemental oxygen requirements, length of time requiring). Enteral feeds (time from operation to first enteral feed, and time to full enteral feeds using the NICU’s calculated goal feed rate), time to first bowel movement, glycerine suppository use and length of stay. For a comprehensive list and definitions of secondary outcomes refer to appendix 1 table 9.

Postoperative pain will be assessed using the pain component of the Neonatal Pain Agitation and Sedation Scale (N-PASS). N-PASS has two scorable components, sedation (measured -10 to 0) and pain (measured 0-10, with up to three points added based on gestational age, therefore 0-13). It includes subjective assessments of behavior and includes objective assessments of vital signs [43]. The N-PASS pain score is validated in term and preterm neonates and for assessment of prolonged and postoperative pain [43, 44]. It is recommended by the Neonatal Pain-Control Group (NPCG) [45]. It has been independently validated and has been shown to be reliable with high clinical utility [46, 47]. The N-PASS pain score is the pain measurement scale currently used in the MCH NICU. The bedside nurses, who will be responsible for measuring pain are therefore comfortable with the use of this instrument, which will increase validity of measurements. Furthermore, documenting this pain score during regular intervals is part of their daily workflow. As this is already required documentation, it is less likely that there will be missing or incomplete study data. It will reduce the burden of documentation required by the beside nurses. An infographic demonstrating the scoring and interpretation of the N-PASS scale can be seen in in Appendix 2, figure 3. initial pain score will be recorded when the patient returns from the operating room. Pain scales will then be done just before the patient’s dose of acetaminophen or placebo (every 4 or 6 hours), as recommended by the NPCG [45]. The neonatologists will have access to the patient’s N-PASS score to aide in clinical decision making. Although staff members on this unit are comfortable with the use an interpretation of this instrument, a table will be available at the beside and distributed to the neonatal physicians and nurses to aide in interpreting N-PASS scores and includes suggested interventions; this is available below in table 4 [48]. Cumulative fentanyl consumption will be calculated in mcg/kg at 24-hour increments, allowing for individual days to be compared and cumulative use throughout the follow up period. The total number of rescue doses per day and at the end of the follow-up period will also be recorded. Any other analgesic or sedative drugs required will be recorded as cumulative doses at 24-hour increments. The requirement for assisted ventilation, including the need for supplemental oxygen, non-invasive mechanical ventilation, or intubation, will be recorded. The length of time of each of these interventions will be recorded during the immediate follow-up period and at the 90-day chart review. Length of stay (with discharge destination) and mortality will also be recorded at the 90-day chart review. Glycerine suppositories were selected for measurement, as they are used commonly in this population due to delayed intestinal function. If there is a difference in the rates of glycerine suppository use, regardless of time to first bowel movement, this may speak to their intestinal function.

Adverse events include vomiting, nasogastric or vygone insertion (number of patients and mean duration), reintubation, apnea, naloxone administration, bradycardia, hypotension, foley catheterization, feeding intolerance, hepatic injury, and mortality.

Vomiting will be recorded if documented in the patient record. Nasogastric or vygone requirements and foley catheterization will be defined as the number of patients and mean duration. Apnea will be defined as the number of patients with an oxygen saturation of less than 94% or respiratory rate of less than 20 breaths per minute for more than 30 seconds. Hypotension will be defined as a systolic blood pressure of less than 60 for more than 30 seconds or requiring vasoactive medications. Bradycardia will be defined as the number of patients with a heart rate less than 100 beats per minute for more than 30 seconds. Feeding intolerance will be defined as the number of patients who had feeds stopped or decreased due to vomiting or increased gastric output, or if diagnosed by the primary team. Hepatic injury will be defined using the same criteria as described in the exclusion criteria. All-cause mortality will be calculated at 90-days.

Tables 8-10 in appendix 1 summarize the secondary outcomes and demographics. Importantly, opioid use during pregnancy and preoperative opioid use for each participant will be capture in the demographic data, in order to help understand the interaction of preoperative opioid use on the outcomes of interest.

| **Table 4.** Pain score interpretation and guidelines for the primary care team | | |
| --- | --- | --- |
| N-PASS Pain Score | Interpretation | Suggested Intervention |
| 0-3 | Normal response for gestational age | - No action - Continue ongoing evaluation |
| 3-13 | Possibility of pain | - Continue ongoing evaluation - Consider increasing analgesia (by increase rate or providing as needed dosing or adding another agent if clinically indicated). |

**6.C Follow Up:** Patients will be followed daily for 192 hours, including the day of surgery (day 0) and the following 7 postoperative days. Post-operative days will be defined based on hours from surgery, as displayed in table 5.

| **Table 5.** Time parameters for postoperative day designation | |
| --- | --- |
| **Post-Operative Day** | **Time From Surgery** |
| Day 0 | 0-23 hours |
| Day 1 | 24-47 hours |
| Day 2 | 48-71 hours |
| Day 3 | 72-95 hours |
| Day 4 | 96-119 hours |
| Day 5 | 120-143 hours |
| Day 6 | 144-167 hours |
| Day 7 | 168-192 hours |

Most neonatal postoperative pain research focuses on the first 48 hours after surgery[19, 49, 50], with a few following patients for 72 hours [50, 51]. A 192-hour follow-up period was selected as this would be more likely to capture the course of severe postoperative pain. This novel data will help tailor pain management strategies at different recovery points. Outcome data (length of stay in the NICU, mortality, length of time requiring invasive ventilation, and need for reintubation) will be extracted from the patient’s chart when they are either discharged from hospital, die, or on postoperative day 90, whichever occurs first. The median length of stay in NICU is variable, especially amongst pre-term infants, with studies estimating 23-219 days [52], with median lengths of stay between 60-70 days [53]. Therefore, 90 days was selected most patients are predicted to be discharged from the NICU. This will be evaluated as part of this feasibility study.

**7.A Sample Size**

A sample size of 30 per group (total of 60 patients) will be used. This decision is based on methodologic guidelines, which suggest that this is an ideal size for assessing feasibility and calculating future sample sizes [54-56].

**7.B** **Interim Analysis and Stopping Guidelines**

A data safety monitoring board (DSMB) will be formed, of three members, including at least one neonatologist and one pediatric surgeon who are not involved in the trial and are unblinded. They will meet when 5 patients have been enrolled and then every six months thereafter. They will meet within fifteen days after a possible study-related (non-life-threatening/non-fatal) adverse event and within 48 hours after a related life-threatening or fatal adverse event is reported. They will create a summary report for the steering committee to categorize adverse events based on severity and relatedness to the study drug. The blinded statistician will prepare the data. An unblinded research assistant will then unblind the data for the DSMB. Furthermore, all adverse events will be reported to the Hamilton Integrated Research Ethics Board (HiREB) and Health Canada. The DSMB will not be able to recommend stopping early for benefit, but may recommend stopping for harm if they observe significant safety concerns.

**8-9 Sequence Generation, Randomization, and Allocation Concealment**

Sequence generation and randomization will be done using REDCap (Research Electronic Data Capture), a secure, web-based software platform designed for study design and data capturing [57]. The randomization code will be generated within REDCap by an unblinded, uninvolved research assistant. Patients will be randomized in a 1:1 fashion between the treatment and control groups. The sequence will be generated in randomly permuted blocks of four or six to ensure equal group sizes. Due to the small sample size, stratification will not be done; however, the balance of demographic features between groups will be evaluated as a feasibility outcome. Patients will be randomized by the PI or research coordinator once their baseline demographic information has been input into REDCap. Each randomized participant will be assigned a randomization number. Using a linking key, the inpatient pharmacy to determine which arm the patient is randomized to. Medication will be prepared and dispensed by a trained research pharmacist. Only the research pharmacists, the unblinded research assistant and DSMB will have access to the randomization key. None of these parties are involved with data collection or analysis.

**10 Implementation**

**10.1** **Enrollment and Randomization**

Patients will be enrolled in the study by members of the research team. Once enrolled, the research team member will input their data into REDCap, then randomizing them and producing a randomization number. The patient’s information and randomization number will be provided to the pharmacy, who will use a randomization key to identify which arm of the trial they are in. They will then prepare the appropriate study drug and will send it to the appropriate patient.

**10.2 Data Collection**

Pain scores (N-PASS) and assessment of blinding will be recorded by the bedside nurse each shift and input into a computer by a study team member. A study team member will collect daily data using the daily data collection form by recording outcomes in the patient’s chart and speaking with the bedside nurse and will complete a daily blinding assessment. The most responsible physician will also complete a daily blinding index. An-end-of-week data collection form will be completed by reviewing previous forms and the patient’s chart. A 90-day data collection form will be completed using the patient’s chart at the end of the 90-day follow-up period.

**10.3 Data Management and Confidentiality**

Study data will be collected and managed using REDCap [57]. Audit trails will be generated with this software. Any paper forms such as consents will be stored in a locked room in the secure office area of the Department of Surgery; only study personnel will have the key to unlock this. Participants will be given a unique study identification number when randomized. The key linking study identification numbers to health record numbers will be kept as an encrypted Microsoft Excel file using AES-256 encryption software software on McMaster’s encrypted OneDrive platform. Identifying data collected in the study key include the patient’s medical record number, sex, date of birth, guardian’s name, and guardian’s contact information (phone, address, and e-mail). Data checks will be completed throughout the trial to ensure the accuracy of all data entered into REDCap. These checks will be completed after subjects 4, 12, 20, with additional checks done randomly throughout the study. Following the trial, paper data will be maintained at McCulloch Office and Storage Systems in Hamilton, Ontario.

All data and information related to the trial will be maintained electronically with encryption and firewall protection, or physically at McCulloch Office and Storage Systems in Hamilton, Ontario for a period of 15 years as required by Health Canada. After the 15 year period de-identified data will be irreversibly destroyed or deleted using shredding for physical data and permanent deletion for electronic data.

**10.4 Direct Access to Source Data/Documentation**

Direct access to source data and documentation will be provided to the Hamilton Integrated Research Ethics Board and to Health Canada for trial related monitoring, audits, review, and inspection.

**10.5 Confidentiality**

**11. Blinding and Unblinding**

Patients, families, researchers, statisticians, nurses, NICU pharmacists and physicians will be blinded to treatments. The McMaster research pharmacists work separately from the NICU pharmacists and will not be blinded but will play no role in analysis or outcome assessment and will only be involved in study drug preparation. As part of randomization, a number will be assigned to the patient. This number will be given to the inpatient pharmacy, where a research pharmacist will compound the medication. In order to prepare the proper treatment, the pharmacist will have a key to determine, based on the number, which arm they were randomized. Saline and IV acetaminophen are indistinguishable when prepared in solution. To ensure the protocol was followed, the key will be checked against the treatments provided at the end of the study. The patient’s chart will not mention which arm of the study the patient was enrolled in, therefore ensuring blinding at the 90-day chart review. The statistician will be provided with blinded data. The groups will not be revealed until after analysis.

If a safety concern is raised by the patient’s physician, member of the research team, the DSMB, the ethics committee, or Health Canada, the research pharmacy will be contacted with the patient’s name and participant number, and they will then provide their allocation. If unblinding occurs, the patient will be discontinued from the study.

**12.A Statistical Analysis**

As this is a feasibility study, all comparative analyses on clinical outcomes are for exploratory purposes, and no inferences will be made from these analyses. Demographics will be reported as means or median for continuous variables and proportion for categorical variables. Feasibility outcomes (recruitment rate, completion rate, discharge rate, success of randomization and protocol violations) will be reported as proportions. Blinding success will be reported with the calculated blinding index, as described by James et al. [42]. Cost will be reported in Canadian dollars for total cost and cost per participant randomized. Secondary clinical outcomes (pain scales, cumulative consumption of fentanyl and other drugs, number of rescue doses, length of time requiring fentanyl infusion, length of time requiring non-invasive (intubation, CPAP, BiPAP, supplemental oxygen), time to enteral fees, time to first bowel movement, and length of stay) will be reported as means or medians with standard deviation and interquartile ranges, respectively. As this study is not adequately powered to detect differences hypothesis testing will not be conducted, but will be conducted for the full scale RCT. Statistics Package for the Social Sciences (SPSS) will be used for data analysis [58].

**12.B Subgroup Analysis**

In the full RCT sub-group analysis is planned for the following demographic variables: gestational age (<32 weeks, ≥ 32 weeks) location of operation (thorax or abdomen), preoperative opioid use, and sex. Aside from sex, these have all been identified as predictors of postoperative pain [59-62]. Gestational age was selected rather than birth weight, as it is a better measure of the infant’s stage of development and a more accurate predictor of physiologic response to pain [63].

Appendix 1: Supplemental Tables

| **Table 6**. Major abdominal and thoracic operations as defined by the Canadian Neonatal Network | |
| --- | --- |
| **Major Abdominal Operations** | **Major Thoracic** |
| Repair or closure of omphalocele | Atrial septal defect closure |
| Repair of aneurysm in internal iliac artery | Blalock-Taussig Shunt (BTS) for tricuspid atresia |
| Closure of bladder rupture | Coarctation repair |
| Bowel resection | Correction of cystic adenomatoid malformation |
| Correction of Atresia | Cystic hygroma |
| Colostomy | Esophageal atresia (thoracic approach) |
| Revision of prolapsing colostomy | Lobectomy |
| Esophageal atresia (abdominal approach) | Lung biopsy (open) |
| Release of corkscrew duodenum | Pacemaker insertion (open) |
| Removal of dermoid cyst (abdominal) | Removal of dermoid cyst (thoracic) |
| Diaphragmatic hernia repair (abdominal approach) | Diaphragmatic hernia repair (thoracic approach) |
| Duodenojejunostomy | Pneumonectomy |
| Fundoplication | Pulmonary artery banding (open) |
| Enterotomy (for removal of meconium) | Pulmonary artery plasty |
| Epispadias repair | Tracheoesophageal (TEF) repair |
| Closure of gastroschisis defect | Vascular ring operation |
| Ileostomy or mucus fistula reversal | Exploratory thoracotomy |
| Ileostomy or mucus fistula creation |  |
| Laparotomy for necrotizing enterocolitis (NEC) |  |
| Nephrectomy |  |
| Omphalomesenteric duct fistula repair |  |
| Orchidectomy |  |
| Pyloromyotomy (open) |  |
| Pyloroplasty |  |
| Vesicostomy closure/revision |  |
| Repair of volvulus |  |
| Exploratory laparotomy |  |

| **Table 7.** Primary Outcomes | | |
| --- | --- | --- |
| **Outcome** | **Definition** | **Success** |
| 1. Recruitment rate | Mean number of patients randomized per month | 2 patients per month |
| 1. Follow up rate | Number of patients followed in completion from postoperative day 0 to 7 | 90% of patients followed completely |
| 1. Medication compliance | Number of patients who received at least 80% of doses of study drugs at the correct dose and interval | 80% |
| 1. Blinding index | Responses of nurse’s physician’s, and research staff’s guess of group assignment (control vs treatment) compared to actual group assignment | Less than 0.20 |

| **Table 8.** Secondary Feasibility Outcomes | |
| --- | --- |
| **Outcome** | **Definition** |
| Time from randomization to start of surgery | Number of hours, positive hours indicate randomized prior to surgery, negative hours indicate randomized after surgery |
| Proportion of eligible patients randomized | Number of patients randomized/number of eligible patients |
| Number of patients with one or more protocol violation | - |
| Cost | Canadian dollars per patient |
| Amount of additional analgesics administered at each pain score in each arm. | Number of patients receiving rescue doses and dose administered (per weight) of additional analgesics for each pain score for pain scores above 14 |

| **Table 9**. Secondary Efficacy Outcomes | |
| --- | --- |
| Outcome | Definition |
| Postoperative Pain | N-PASS pain score every four or six hours, filled out by RN |
| Fentanyl consumption | Cumulative over 24/hour periods, over entire study period, and duration of fentanyl infusion |
| Consumption of other analgesics | Cumulative over 24/hour periods and over entire study period |
| Invasive ventilation | Length of time requiring intubation |
| Non-invasive ventilation | Length of time requiring CPAP, BiPAP, or supplemental oxygen |
| Enteral feeds | Time to first enteral feeds and time to full enteral feeds (using NICU’s calculated goal feed) |
| Bowel movement | Time to first bowel movement |
| Glycerin suppository use | Number of patients requiring one or more glycerin suppositories |
| Length of stay | At 90-day chart review (with discharge destination) |

| **Table 10.** Secondary Adverse Event Outcomes | |
| --- | --- |
| Outcome | Definition |
| Vomiting | Number of patients with ≥ 1 episode of vomiting documented |
| NG/Vygone | Number of patients, mean duration |
| Reintubation | Number of patients |
| Apnea | Number of patients with oxygen saturation less than 94% or RR less than 20 breaths/min for more than 30 seconds |
| Naloxone administration | Number of patients |
| Bradycardia | Number of patients with HR less than 100 for more than 30 seconds |
| Hypotension | Number of patients with SBP less than 60, or requiring vasoactive medication |
| Foley catheterization | Number of patients, mean duration |
| Feeding intolerance | Number of patients: feeds stopped or decreased due to vomit/increased gastric output, or if diagnosed by the treating team |
| Hepatic injury | Number of patients |
| Mortality | All-cause mortality at 90 days |

| **Table 11.** Demographic Data |
| --- |
| 1. Sex |
| 1. Race |
| 1. Gestational age |
| 1. Birth weight |
| 1. Age at surgery |
| 1. Preoperative diagnosis |
| 1. Procedure |
| 1. Length of OR |
| 1. Preoperative opioid use (administered opioids within 7 days prior to surgery) |
| 1. Intubated preoperatively |
| 1. Maternal opioid use (use of opioids on more than two separate days during pregnancy) |

| **Table 12.** Summary of acceptable formulations and concentrations of fentanyl for use in this trial | |
| --- | --- |
| **Brands** | **Prepared syringe concentrations** |
| SteriMax Fentanyl (fentanyl citrate injection) 100 mcg/2 mL Ampule (Concentration: 50 mcg/mL) DIN 02496143 | Fentanyl 1000 mcg/50 mL of D5W (or normal saline or D10W) syringe (concentration: 20 mcg/mL) |
| SteriMax Fentanyl (fentanyl citrate injection) 250 mcg/5 mL Vial (Concentration: 50 mcg/mL) DIN 02496151 | Fentanyl 250 mcg/50 mL D5W (or normal saline or D10W) syringe (concentration: 5 mcg/mL) |
| Sandoz Fentanyl (fentanyl citrate injection) 100 mcg/2 mL Ampule & 250 mcg/5 mL vial (Concentration: 50 mcg/mL) DIN 02240434 | Fentanyl 50 mcg/50 mL D5W (or normal saline or D10W) syringe (concentration: 1 mcg/mL) |
| Sandoz Fentanyl (fentanyl citrate injection) 100 mcg/2 mL Ampule (Concentration: 50 mcg/mL) DIN 02384124 |  |
|  |  |

Appendix 2: Supplemental Figures

**Figure 2.** McMaster Children’s Hospital fentanyl formulation instructions.


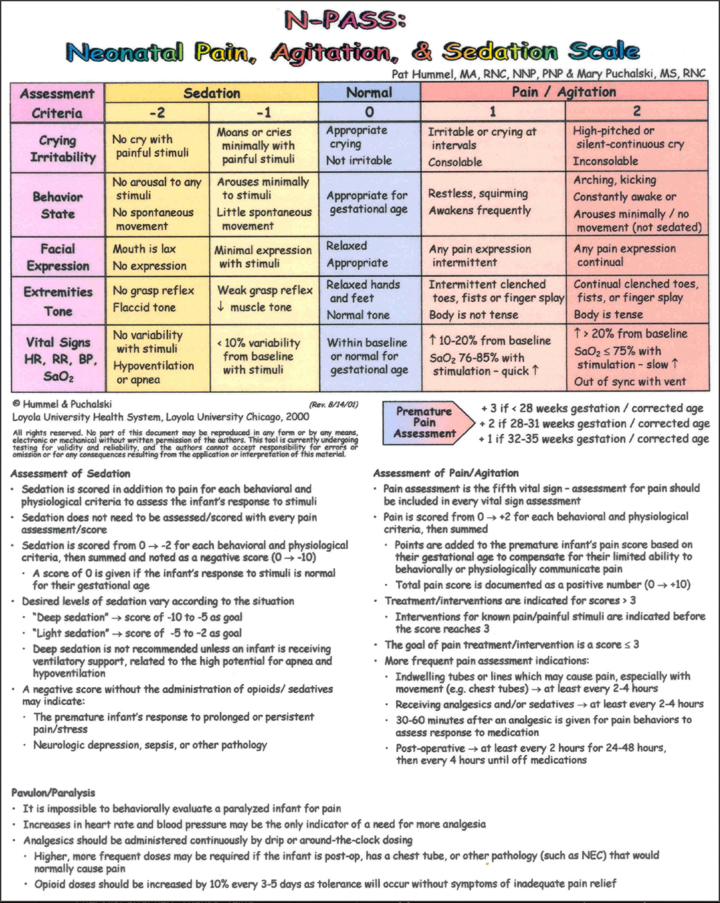


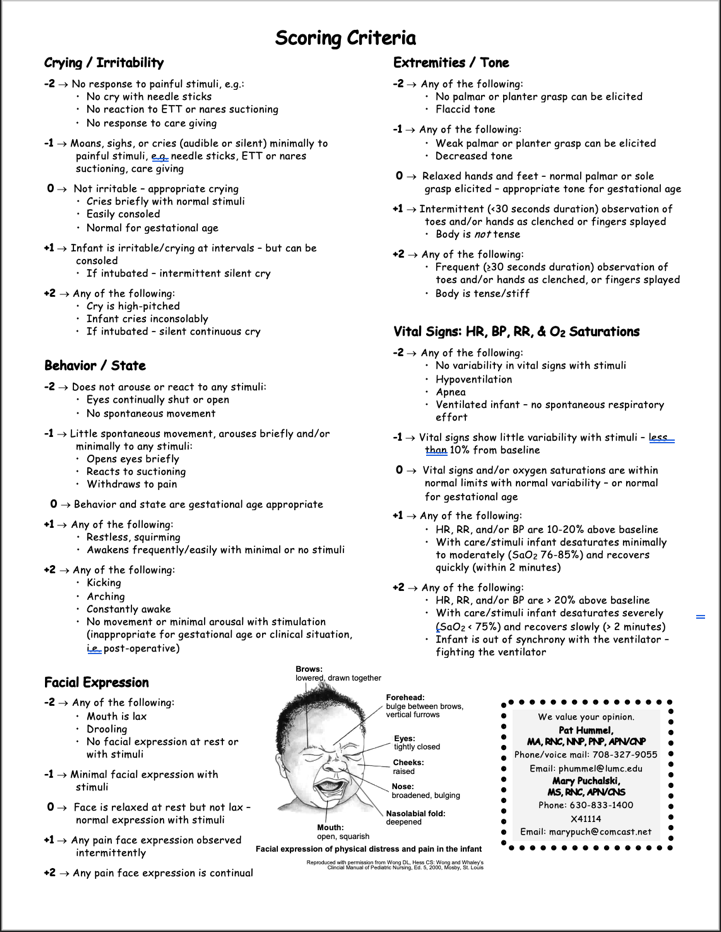


**Figure 3.** N-PASS scoring sheet and interpretation instructions

References

1. Yaster, M., P.P. McNaull, and P.J. Davis, *The opioid epidemic in pediatrics: a 2020 update.* Current Opinion in Anesthesiology, 2020. **33**(3): p. 327-334.

2. Winstanley, E.L. and A.N. Stover, *The Impact of the Opioid Epidemic on Children and Adolescents.* Clinical Therapeutics, 2019. **41**(9): p. 1655-1662.

3. Daley, D.C., et al., *Forgotten but not gone: The impact of the opioid epidemic and other substance use disorders on families and children.* Commonwealth, 2018. **20**(2-3).

4. Mak, W.Y., et al., *Pharmacotherapy for acute pain in children: current practice and recent advances.* Expert opinion on pharmacotherapy, 2011. **12**(6): p. 865-881.

5. Chiaretti, A., et al., *Current practice and recent advances in pediatric pain management.* Eur Rev Med Pharmacol Sci, 2013. **17**(Suppl 1): p. 112-126.

6. Brasher, C., et al., *Postoperative Pain Management in Children and Infants: An Update.* Pediatric Drugs, 2014. **16**(2): p. 129-140.

7. Swegle, J.M. and C.D. Logemann, *Management of common opioid-induced adverse effects.* American family physician, 2006. **74**(8): p. 1347-1354.

8. Pizzi, L.T., et al., *Relationship between potential Opioid‐Related adverse effects and hospital length of stay in patients receiving opioids after orthopedic surgery.* Pharmacotherapy: The Journal of Human Pharmacology and Drug Therapy, 2012. **32**(6): p. 502-514.

9. Attarian, S., et al., *The Neurodevelopmental Impact of Neonatal Morphine Administration.* Brain Sciences, 2014. **4**(2): p. 321-334.

10. Puia-Dumitrescu, M., et al., *Assessment of 2-Year Neurodevelopmental Outcomes in Extremely Preterm Infants Receiving Opioids and Benzodiazepines.* JAMA Network Open, 2021. **4**(7): p. e2115998-e2115998.

11. Bosenberg, A. and R.P. Flick, *Regional anesthesia in neonates and infants.* Clinics in perinatology, 2013. **40**(3): p. 525-538.

12. Fitzgerald, M. and S. Beggs, *Book Review: The neurobiology of pain: Developmental aspects.* The Neuroscientist, 2001. **7**(3): p. 246-257.

13. Maxwell, L.G., C.P. Malavolta, and M.V. Fraga, *Assessment of pain in the neonate.* Clinics in perinatology, 2013. **40**(3): p. 457-469.

14. Arana, A., N.S. Morton, and T.G. Hansen, *Treatment with paracetamol in infants.* Acta Anaesthesiologica Scandinavica, 2001. **45**(1): p. 20-29.

15. Shastri, N., *Intravenous Acetaminophen Use in Pediatrics.* Pediatric Emergency Care, 2015. **31**(6): p. 444-448.

16. Zuppa, A.F., et al., *Safety and population pharmacokinetic analysis of intravenous acetaminophen in neonates, infants, children, and adolescents with pain or Fever.* The journal of pediatric pharmacology and therapeutics : JPPT : the official journal of PPAG, 2011. **16**(4): p. 246-261.

17. Terrin, G., et al., *Paracetamol for the treatment of patent ductus arteriosus in preterm neonates: a systematic review and meta-analysis.* Archives of Disease in Childhood-Fetal and Neonatal Edition, 2016. **101**(2): p. F127-F136.

18. Ceelie, I., et al., *Effect of Intravenous Paracetamol on Postoperative Morphine Requirements in Neonates and Infants Undergoing Major Noncardiac Surgery A Randomized Controlled Trial.* Jama-Journal of the American Medical Association, 2013. **309**(2): p. 149-154.

19. Dehghan, K., et al., *The Comparison of the Analgesic Effect of Intravenous Acetaminophen with Fentanyl in Thoracic and Abdominal Surgeries of Newborns.* International Journal of Pediatrics-Mashhad, 2019. **7**(7): p. 9773-9781.

20. Hong, J.-Y., et al., *Fentanyl-sparing Effect of Acetaminophen as a Mixture of Fentanyl in Intravenous Parent-/Nurse-controlled Analgesia after Pediatric Ureteroneocystostomy.* Anesthesiology, 2010. **113**(3): p. 672-677.

21. Solanki, N., S. Engineer, and P. Vecham, *Comparison of epidural versus systemic analgesia for major surgeries in neonates and infants.* Journal of clinical neonatology, 2017. **6**(1): p. 23‐28.

22. Murat, I., et al., *Tolerance and analgesic efficacy of a new i.v. paracetamol solution in children after inguinal hernia repair.* Pediatric Anesthesia, 2005. **15**(8): p. 663-670.

23. Network, C.N. *The Canadian Neonatal Network Abstractor’s Guide*. 2015 [cited 2022 February 10]; 2.2:[Available from: <http://www.canadianneonatalnetwork.org/portal/Portals/0/CNN%20Manuals/CNN%20Manual_20150814.pdf>.

24. Davidson, J.M., et al., *A randomized trial of intravenous acetaminophen versus indomethacin for treatment of hemodynamically significant PDAs in VLBW infants.* Journal of Perinatology, 2021. **41**(1): p. 93-99.

25. Palmer, G.M., et al., *I.V. acetaminophen pharmacokinetics in neonates after multiple doses.* British Journal of Anaesthesia, 2008. **101**(4): p. 523-530.

26. Squires, R.H., et al., *Acute liver failure in children: The first 348 patients in the pediatric acute liver failure study group.* The Journal of Pediatrics, 2006. **148**(5): p. 652-658.e2.

27. Taylor, S.A. and P.F. Whitington, *Neonatal acute liver failure.* Liver Transpl, 2016. **22**(5): p. 677-85.

28. Ciocca, M. and F. Álvarez, *Neonatal acute liver failure: a diagnosis challenge.* Arch Argent Pediatr, 2017. **115**(2): p. 175-180.

29. Palevsky, P.M., et al., *KDOQI US commentary on the 2012 KDIGO clinical practice guideline for acute kidney injury.* American Journal of Kidney Diseases, 2013. **61**(5): p. 649-672.

30. Childs, C. and R.A. Little, *Acetaminophen (paracetamol) in the management of burned children with fever.* Burns, 1988. **14**(5): p. 343-348.

31. Pacifici, G.M. and K. Allegaert, *Clinical pharmacology of paracetamol in neonates: a review.* Current therapeutic research, clinical and experimental, 2014. **77**: p. 24-30.

32. Allegaert, K., I. Murat, and B.J. Anderson, *Not all intravenous paracetamol formulations are created equal.* Paediatr Anaesth, 2007. **17**(8): p. 811-2.

33. Allegaert, K., G.M. Palmer, and B.J. Anderson, *The pharmacokinetics of intravenous paracetamol in neonates: size matters most.* Arch Dis Child, 2011. **96**(6): p. 575-80.

34. Bartocci, M. and S. Lundeberg, *Intravenous paracetamol: the 'Stockholm protocol' for postoperative analgesia of term and preterm neonates.* Paediatric Anaesthesia, 2007. **17**(11): p. 1120-1.

35. Lexicomp Inc. *Acetaminophen (paracetamol): Pediatric drug information*. 2022 2022 [cited 2022 June]; Available from: <https://www.uptodate.com/contents/acetaminophen-paracetamol-pediatric-drug-information?search=acetaminophen-paracetamol-drug-&topicRef=9242&source=see_link>.

36. Howie, S.R., *Blood sample volumes in child health research: review of safe limits.* Bull World Health Organ, 2011. **89**(1): p. 46-53.

37. Sisson, T.R.C., L.E. Whalen, and A. Telek, *The blood volume of infants: II. The premature infant during the first year of life.* The Journal of Pediatrics, 1959. **55**(4): p. 430-446.

38. Lau, W. *Chapter 13 Neonatal and Pediatric Transfusions*. Canadian Blood Services Tranfusion Clinical Guide 2017 August 2 2017 [cited 2022; Available from: <https://professionaleducation.blood.ca/en/transfusion/clinical-guide/neonatal-and-pediatric-transfusion>.

39. World Health Organization, *WHO Guidelines Approved by the Guidelines Review Committee*, in *WHO Guidelines on the Pharmacological Treatment of Persisting Pain in Children with Medical Illnesses*. 2012, World Health Organization: Geneva.

40. Anand, K.J., *Consensus statement for the prevention and management of pain in the newborn.* Arch Pediatr Adolesc Med, 2001. **155**(2): p. 173-80.

41. Bosworth, H.B., *Medication Adherence*, in *Improving Patient Treatment Adherence: A Clinician's Guide*, H. Bosworth, Editor. 2010, Springer New York: New York, NY. p. 68-94.

42. James, K.E., et al., *An index for assessing blindness in a multi-centre clinical trial: disulfiram for alcohol cessation--a VA cooperative study.* Stat Med, 1996. **15**(13): p. 1421-34.

43. Hummel, P., et al., *Clinical reliability and validity of the N-PASS: neonatal pain, agitation and sedation scale with prolonged pain.* J Perinatol, 2008. **28**(1): p. 55-60.

44. Hummel, P., P. Lawlor-Klean, and M.G. Weiss, *Validity and reliability of the N-PASS assessment tool with acute pain.* J Perinatol, 2010. **30**(7): p. 474-8.

45. Anand, K.J.S., et al., *Summary Proceedings From the Neonatal Pain-Control Group.* Pediatrics, 2006. **117**(Supplement_1): p. S9-S22.

46. Desai, A., et al., *Comparing N-PASS and NIPS: improving pain measurement in the neonate.* Advances in Neonatal Care, 2018. **18**(4): p. 260-266.

47. Benbrook, K., et al., *Agreement of the Neonatal Pain, Agitation, and Sedation Scale (N-PASS) With NICU Nurses’ Assessments.* Advances in Neonatal Care, 2022: p. 10.1097.

48. Lyngstad, L.T., S. Steinnes, and F. Le Marechal, *Improving pain management in a neonatal intensive care unit with single-family room—A quality improvement project.* Paediatric and Neonatal Pain, 2022. **4**(2): p. 69-77.

49. Ceelie, I., et al., *Effect of intravenous paracetamol on postoperative morphine requirements in neonates and infants undergoing major noncardiac surgery: a randomized controlled trial.* JAMA, 2013. **309**(2): p. 149-54.

50. Hong, J.-Y., et al., *Fentanyl Sparing Effects of Combined Ketorolac and Acetaminophen for Outpatient Inguinal Hernia Repair in Children.* Journal of Urology, 2010. **183**(4): p. 1551-1555.

51. Hong, J.-Y., et al., *Paracetamol Reduces Postoperative Pain and Rescue Analgesic Demand After Robot-Assisted Endoscopic Thyroidectomy by the Transaxillary Approach.* World Journal of Surgery, 2010. **34**(3): p. 521-526.

52. Lee, H.C., et al., *Accounting for variation in length of NICU stay for extremely low birth weight infants.* Journal of Perinatology, 2013. **33**(11): p. 872-876.

53. Tawfik, D.S., et al., *Safety climate, safety climate strength, and length of stay in the NICU.* BMC Health Services Research, 2019. **19**(1): p. 738.

54. Billingham, S.A., A.L. Whitehead, and S.A. Julious, *An audit of sample sizes for pilot and feasibility trials being undertaken in the United Kingdom registered in the United Kingdom Clinical Research Network database.* BMC Med Res Methodol, 2013. **13**: p. 104.

55. Browne, R.H., *On the use of a pilot sample for sample size determination.* Stat Med, 1995. **14**(17): p. 1933-40.

56. Thabane, L., et al., *A tutorial on pilot studies: the what, why and how.* BMC Medical Research Methodology, 2010. **10**(1): p. 1.

57. Harris, P.A., et al., *The REDCap consortium: Building an international community of software platform partners.* Journal of Biomedical Informatics, 2019. **95**: p. 103208.

58. IBM®, *SPSS® Statistics*. 2019.

59. Kalkman, C.J., et al., *Preoperative prediction of severe postoperative pain.* Pain, 2003. **105**(3): p. 415-423.

60. Mekonnen, Z.A., et al., *Prevalence and Contributing Factors Associated With Postoperative Pain in Pediatric Patients: A Cross-Sectional Follow-up Study.* Perioperative Care and Operating Room Management, 2021. **23**: p. 100159.

61. Ancora, G., et al., *Influence of gestational age on the EDIN score: an observational study.* Archives of Disease in Childhood - Fetal and Neonatal Edition, 2009. **94**(1): p. F35-F38.

62. Caljouw, M.A.A., et al., *Measurement of pain in premature infants with a gestational age between 28 to 37 weeks: Validation of the adapted COMFORT scale.* Journal of Neonatal Nursing, 2007. **13**(1): p. 13-18.

63. Wilcox, A.J., *On the importance—and the unimportance— of birthweight.* International Journal of Epidemiology, 2001. **30**(6): p. 1233-1241.
