# Supplementary material for: Intravenous acetaminophen for postoperative pain in the neonatal intensive care unit: A protocol for a pilot randomized controlled trial (IVA POP)
Source: PLoS One. 2023 Nov 20;18(11):e0294519. doi: 10.1371/journal.pone.0294519 (PMC10659208; doi:10.1371/journal.pone.0294519)
Supplement: S1 Table — (DOCX) [file pone.0294519.s003.docx]

| **S1 Table**. Major abdominal and thoracic operations as defined by the Canadian Neonatal Network | |
| --- | --- |
| **Major Abdominal Operations** | **Major Thoracic** |
| Repair or closure of omphalocele | Atrial septal defect closure |
| Repair of aneurysm in internal iliac artery | Blalock-Taussig Shunt (BTS) for tricuspid atresia |
| Closure of bladder rupture | Coarctation repair |
| Bowel resection | Correction of cystic adenomatoid malformation |
| Correction of Atresia | Cystic hygroma |
| Colostomy | Esophageal atresia (thoracic approach) |
| Revision of prolapsing colostomy | Lobectomy |
| Esophageal atresia (abdominal approach) | Lung biopsy (open) |
| Release of corkscrew duodenum | Pacemaker insertion (open) |
| Removal of dermoid cyst (abdominal) | Removal of dermoid cyst (thoracic) |
| Diaphragmatic hernia repair (abdominal approach) | Diaphragmatic hernia repair (thoracic approach) |
| Duodenojejunostomy | Pneumonectomy |
| Fundoplication | Pulmonary artery banding (open) |
| Enterotomy (for removal of meconium) | Pulmonary artery plasty |
| Epispadias repair | Tracheoesophageal (TEF) repair |
| Closure of gastroschisis defect | Vascular ring operation |
| Ileostomy or mucus fistula reversal | Exploratory thoracotomy |
| Ileostomy or mucus fistula creation |  |
| Laparotomy for necrotizing enterocolitis (NEC) |  |
| Nephrectomy |  |
| Omphalomesenteric duct fistula repair |  |
| Orchidectomy |  |
| Pyloromyotomy (open) |  |
| Pyloroplasty |  |
| Vesicostomy closure/revision |  |
| Repair of volvulus |  |
| Exploratory laparotomy |  |
